# Supplementary figures and images for: Predicting mitochondrial fission, fusion and depolarisation event locations from a single z-stack
Source: PLoS One. 2023 Mar 8;18(3):e0271151. doi: 10.1371/journal.pone.0271151 (PMC9994753; doi:10.1371/journal.pone.0271151)

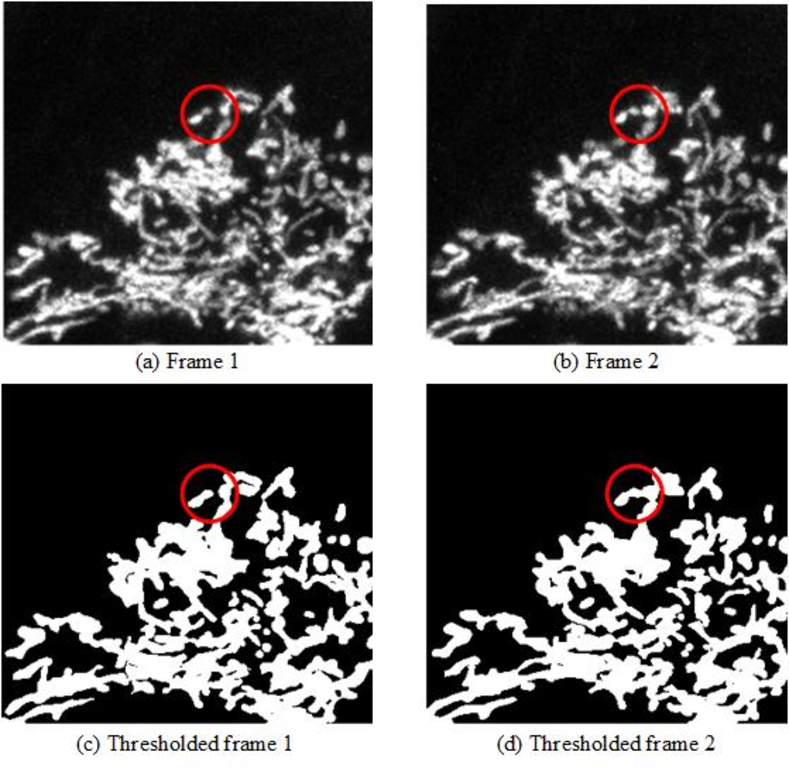

Supplement: S1 Fig — Two frames (a and b) of a time-lapse sequence show structures which would incorrectly be classified by MEL as fusion candidates, highlighted with red circles. The thresholded versions of these two frames are shown in (c) and (d). MEL would incorrectly localise this event due to the binarisation of the low intensity bridge between the structures. (TIF) [file pone.0271151.s001.tif]

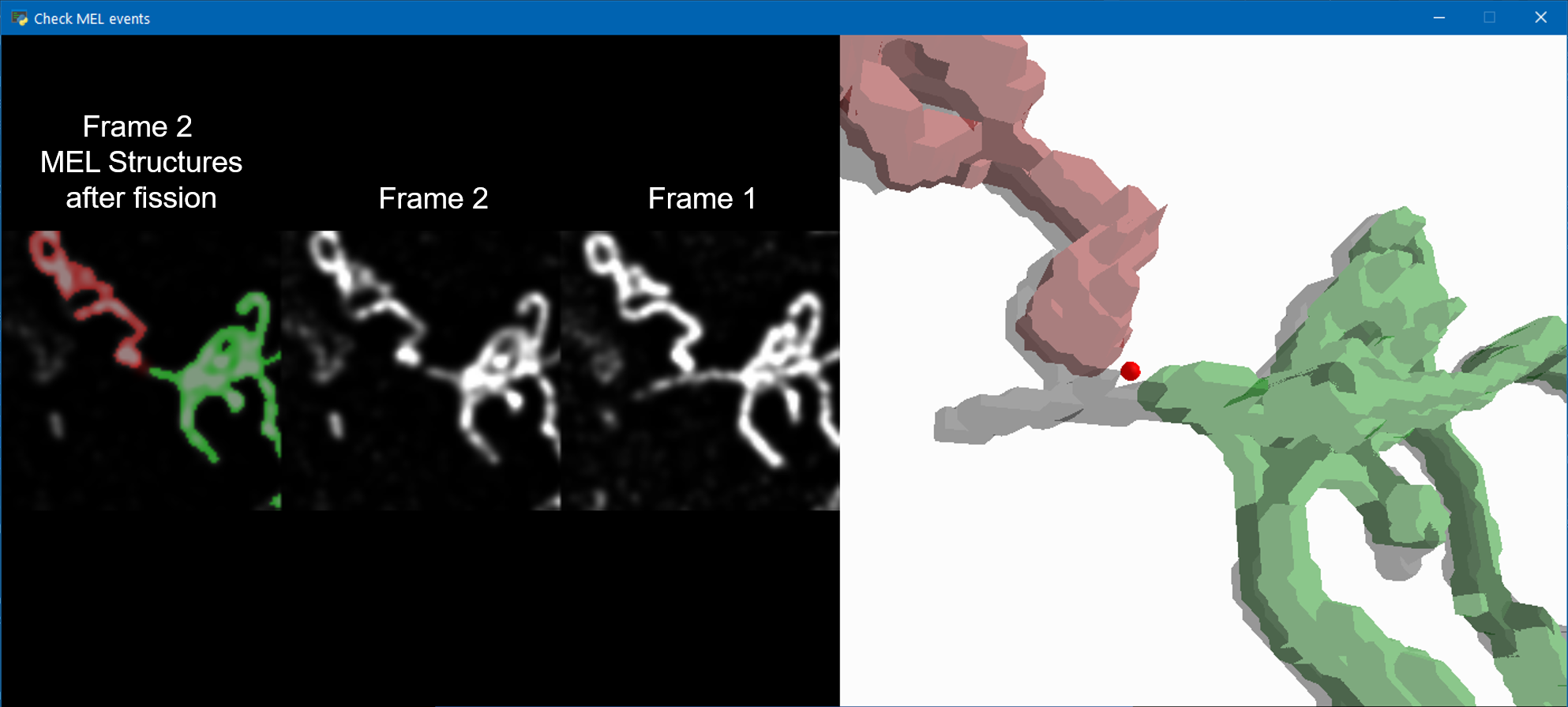

Supplement: S2 Fig — An image of the post MEL process event validation tool used to validate the legitimacy of events, where a fission event was analysed. In the left panel the two structures in Frame2 that underwent fission are shown in red and green. The deconvolved Frames1 and 2 are then shown to assist the user in determining whether the event is real. The panel on the right shows a three-dimensional model of the red and green structures in Frame2 overlayed on the original structure in Frame1 (grey structure). The fission event is indicated by the red dot. Reproduced form Theart et al., [18]. (TIF) [file pone.0271151.s002.tif]

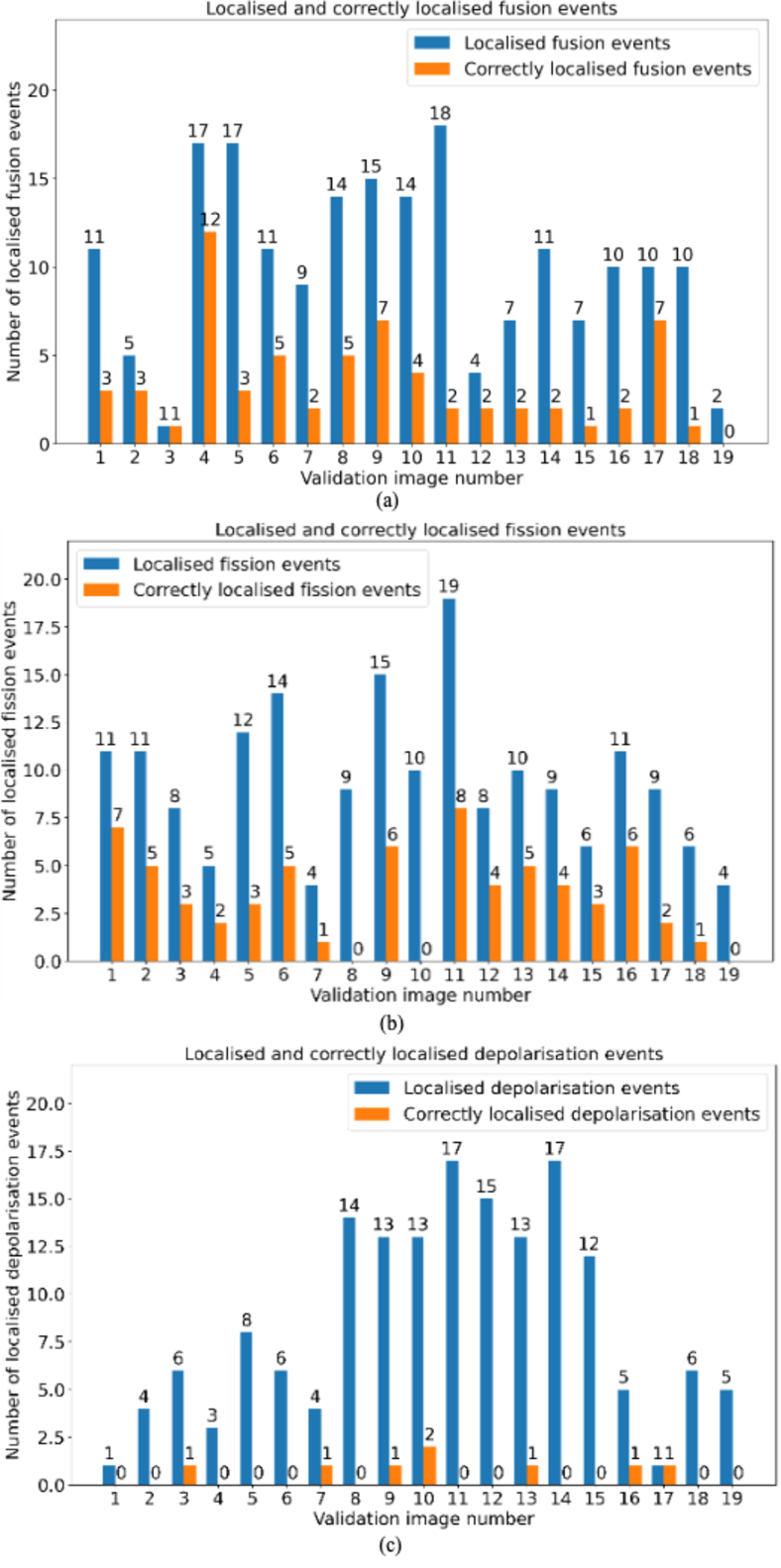

Supplement: S3 Fig — Predicted and correctly predicted mitochondrial fusion (a), fission (b) and depolarisation (c) events by the Pix2Pix GAN for the 19 validation images used during training. (TIF) [file pone.0271151.s003.tif]

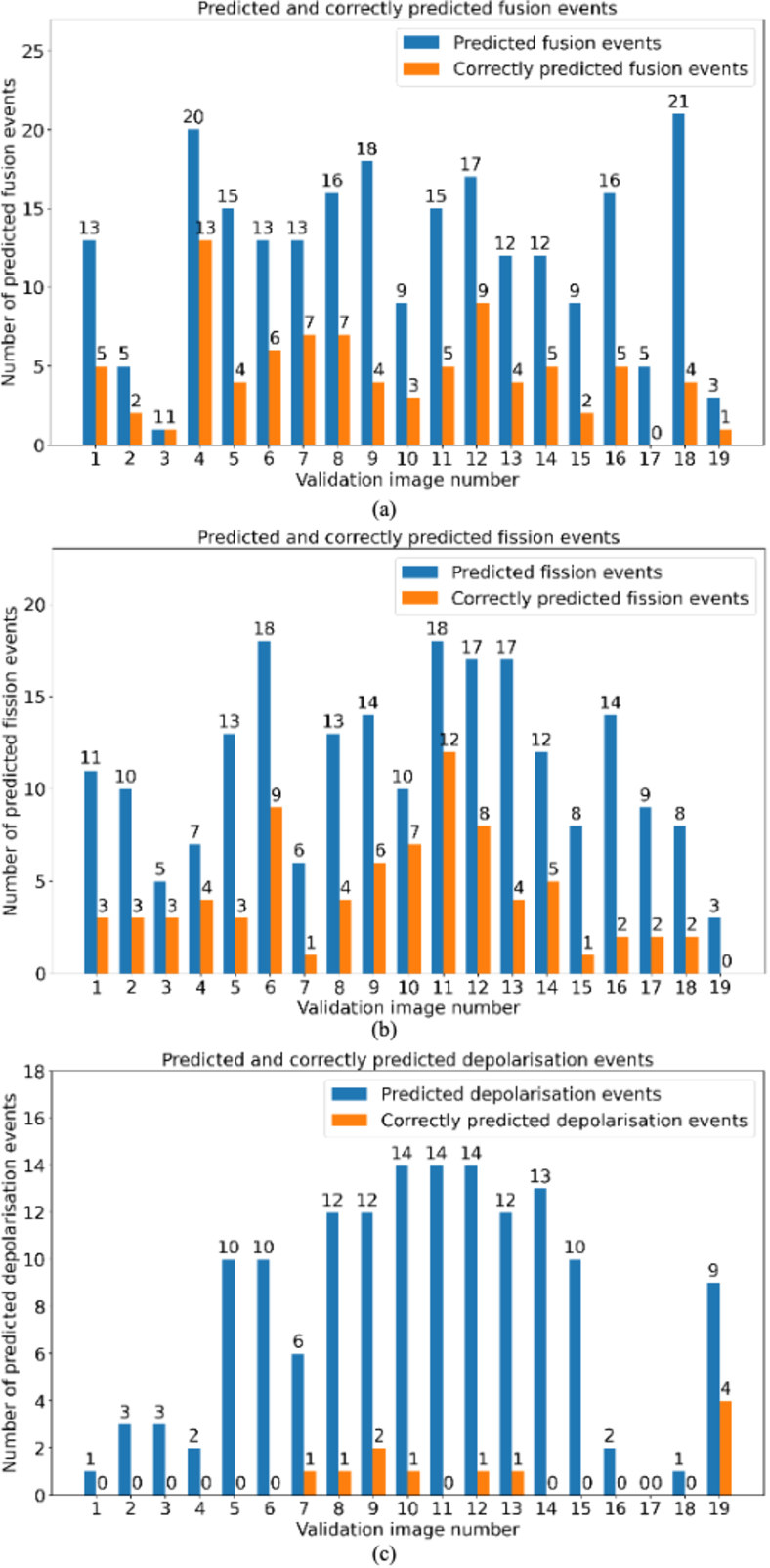

Supplement: S4 Fig — Predicted and correctly predicted mitochondrial fusion (a), fission (b) and depolarisation (c) events by the Vox2Vox GAN for the 19 validation images used during training. (TIF) [file pone.0271151.s004.tif]
