# Supplementary material for: Predicting mitochondrial fission, fusion and depolarisation event locations from a single z-stack
Source: PLoS One. 2023 Mar 8;18(3):e0271151. doi: 10.1371/journal.pone.0271151 (PMC9994753; doi:10.1371/journal.pone.0271151)
Supplement: Attachment — Submitted filename: Response to Reviewers.pdf [file pone.0271151.s005.pdf]

Response to the reviewers of the submission titled: Three-Dimensional Mitochondrial Fission, Fusion and Depolarisation Event Location Prediction for a High-Throughput Analysis of Fluorescence Microscopy Images
by J.G. de Villiers and R.P. Theart

I would like to thank the reviewers for their thorough review of the submission. Your comments are invaluable and assisted me in producing a much better final document.

Reviewer 1

Reviewer's comment 1:

> In the paper the prediction of occurrence and exact locations of mitochondrial fission, fusion and depolarisation events in three dimensions by using two types of a generative adversarial network (GAN) is presented. The strength of the paper is the innovative application of the GAN network to the aforementioned prediction. On the other hand, the weakness of the work is that both types of applied GAN networks achieved worse results than the MEL used so far. Nevertheless, due to the innovative approach to the problem, as well as the prospect of improving the described approach, the journal may be published in PLOS One.

Response comment 1:

> I would like to thank the reviewer for pointing out ambiguity in the submission. The MEL method receives two sequential frames of a time-lapse sequence as input. It then compares these two frames to determine the locations of mitochondrial fission, fusion and depolarisation events. Therefore, MEL localises these events based on two frames. The GANs discussed in this submission, on the other hand, receives only a single frame of a time-lapse sequence as input and is tasked with predicting the locations of mitochondrial events. Consequently, the GANs have far less information at their disposal and consequently, the fact that they achieved comparable accuracy to the MEL method is a very positive results indicating that the current morphology of the mitochondria contains a significantly amount of information to aid this prediction. In an effort to reduce this ambiguity a specific reference to MEL using two sequential frames of a time-lapse sequence as input has been added to the introduction and related work sections of the submission.

Reviewer 2

Reviewer's comment 1:

> The authors have not mentioned what's the motivation and challenges. Such as "This paper documents the development of a novel method to predict the occurrence and exact locations of mitochondrial fission, fusion and depolarisation events in three dimensions" But why?

Response comment 2:

> I appreciate the reviewer pointing out this lack of information in the submission. The following paragraph was added to the abstract:
>
> "This novel implementation of neural networks to predict these events using information encoded only in the morphology of the mitochondria eliminate the need for time-lapse sequences of cells. The ability to predict these morphological mitochondrial events using a single image can not only democratise research but also revolutionise drug trials."

Reviewer's comment 2:

In this paper the author used two types of network. Such as "These occurrence and location of these events were successfully predicted with a three-dimensional version of the Pix2Pix generative adversarial network (GAN) as well as a three-dimensional adversarial segmentation network called the Vox2Vox GAN.". Here again a question rises that why the authors used this network

Response comment 2:

I would like to thank the reviewer for this comment. In an attempt to explain the use of these networks the following paragraph was added to the introduction.
"The ability to predict the locations of these events on a z-stack image using only a single image and information encoded only in the morphology of the mitochondria implied that generative networks were needed. For this reason generative neural networks were used. These networks were trained using pairs of input and desired output images. To our knowledge the use of generative networks to predict the locations of mitochondrial events has never been done before."

Reviewer's comment 3:

I have not found any details of methodology in introduction. Briefly describe the methodology along with motivation, challenges and related work.

Response comment 3:

I would like to thank the reviewer for pointing out this omission in the submission. I have added the following to my Introduction:

"Until recently, the occurrence and locations of these events were determined through manual visual inspection by comparing two frames of a time-lapse sequence. Recently, an automatic method for comparing two frames of a time-lapse sequence to localise these events has been developed. This automatic method was used to generate the training data for the neural networks discussed in this paper. These networks use a single frame of a time-lapse sequence as input and makes predictions using information encoded in the morphology of the mitochondria.
Mitochondrial event predictions using neural networks enable insight into the health of cells from a single z-stack, thereby eliminating the need to capture time-lapse sequences. The removal of time-lapse sequences from the analysis process enables the use of fixed cells rather than live cells while still obtaining similar analysis insights. Furthermore, neural networks democratise this type of research by providing researchers who have no access to the equipment necessary to obtain time-lapse sequences with the opportunity to conduct similar studies."

Reviewer's comment 4:

Related work section shows a detailed story of various existing methods. The authors should connect these stories with the proposed work. Second, why a separate section for each method?

Response comment 4:

I would like to thank the reviewer for pointing out this error in my submission The two GANs are now discussed in the same section and the following paragraph was added at the start of the section.

"To our knowledge, no other methods capable of predicting these events exist and as a consequence this section discusses the automatic method that was is used for localising mitochondrial fission, fusion and depolarisation events by comparing two frames of a time-lapse sequence, which was used to generate the ground truth data for the neural networks discussed in this paper. This section also discusses the most prominent generative neural networks used in industry."

Reviewer's comment 5:

The heading "Event location penalised MAE loss (ELP-MAE)" is quite confusing. Please enhance it.

Response comment 5:

I would like to thank the reviewer for pointing out this point of confusion in the submission. The heading of the section has been changed to the following:
"Location based weighted mean absolute error loss function"

Reviewer's comment 6:

It is better not to use abbreviations in the heading. "Comparison of results with MEL accuracies". Also, the author should compare the proposed with at least three exiting methods.

Response comment 6:

I would like to thank the reviewer for this comment. All abbreviations were removed from the headings. Unfortunately, MEL is to our knowledge the only method currently capable of automatically localising mitochondrial events in three dimensions. Furthermore, the use of GANs to predict the locations of these events are also, to our knowledge, the first of their kind and we can thus not compare them to any other methods.

Reviewer's comment 7:

The title of the paper is very ambiguous. Use a more concise title.

Response comment 7:

I would like to thank the reviewer for this comment. The title has been changed to the following:
"Predicting mitochondrial fission, fusion and depolarisation event locations from a single z-stack"
