# Supplementary material for: Predicting mitochondrial fission, fusion and depolarisation event locations from a single z-stack
Source: PLoS One. 2023 Mar 8;18(3):e0271151. doi: 10.1371/journal.pone.0271151 (PMC9994753; doi:10.1371/journal.pone.0271151)
Supplement: Attachment — Submitted filename: Response to Reviewers.pdf [file pone.0271151.s006.pdf]

Response to the reviewers of the submission titled: Predicting mitochondrial fission, fusion and depolarisation event locations from a single z-stack
by J.G. de Villiers and R.P. Theart

We would like to thank the reviewers for their thorough review of the submission. Your comments are invaluable and assisted us in producing a much better final document.

Reviewer #3

Reviewer's comment 1:

> TMRE is membrane potential dependent chemical dye. The manuscript shall address this issue in the discussion. If the authors could apply membrane potential insensitive dye or fluorescence protein targeting to mitochondria to perform the prediction under different stress condition (with/without CCCP), the readers will be much more appreciated.

Response comment 1:

> We would like to thank the reviewer for pointing out this possible source of gaining better reader satisfaction.
>
> We have now updated the the Materials and Methods section to clarify our justification for using TMRE, by stating "TMRE is used widely when assessing mitochondrial dysfunction and has a good signal-to-noise ratio. Additionally, since TMRE is a membrane potential dependent chemical dye it allows us to create the ground truth data for mitochondrial depolarisation which MEL is able to detect." Furthermore, since the MEL method, on which this new work is based, also used TMRE in the development, it was deemed to provide the most direct comparison with that method. However, since we argue that our method primarily rely on the morphology of the mitochondrial network, future work should be able to use other mitochondrial probes, such as MitoTracker, and achieve similar result.

Reviewer's comment 2:

> The cause-effect relation between mtDNA and mitophagy described in line 7-8 is controversy.

Response comment 2:

> We appreciate the reviewer pointing out this point of controversy in the article. Indeed the link between mitochondrial events and mitophagy is controversial. However, there are articles documenting a link between mtDNA and mitophagy. References to these articles have been added to the article:
>
> "To reduce the risk of mtDNA mutation accumulation, a mitochondrion can undergo dynamic events such as fission, fusion and mitochondrial autophagy (mitophagy) [1, 2]."
>
> Furthermore, since this is only provided for some context and motivation for the study in this paper, and not part of the main study itself, no additional changes was deemed necessary.

Reviewer's comment 3:

The description of membrane depolarization and ROS in line 18-19 is also controversy.

Response comment 3:

We appreciate the reviewer for pointing out this point of controversy in the article. We have clarified this as shown below. Furthermore, since this is only provided for some context and motivation for the study in this paper, and not part of the main study itself, no additional changes was deemed necessary.

The following was added to to the Introduction:
The mitochondrial respiratory chain is the main site of cellular ROS production, while mitochondria are also highly sensitive to, and affected by, ROS toxicity [3]. ROS induced ROS release (RIRR) has been described as a positive feedback mechanism that is involved in the interaction between ROS and the mitochondria where a surge of mitochondrial ROS is induced by ROS, subsequently causing a reduction in the mitochondrial membrane potential [4]. For this reason mitochondrial depolarisation is required for mitochondrial quality control and removal of dysfunctional mitochondria through mitophagy.

References

[1] Parone PA, Druz SD, Tondera D, Mattenberger Y, James DI, Maechler P, et al. Preventing mitochondrial fission impairs mitochondrial function and leads to loss of mitochondrial DNA. PLoS ONE. 2008;3.

[2] Mourier A, Motori E, Brandt T, Lagouge M, Atanassov I, Galinier A, et al. Mitofusin 2 is required to maintain mitochondrial coenzyme Q levels. Journal of Cell Biology. 2015;208.

[3] Wu Y, Chen M, Jiang J. Mitochondrial dysfunction in neurodegenerative diseases and drug targets via apoptotic signaling. Mitochondrion. 2019 11;49:35-45.

[4] Fang C, Gu L, Smerin D, Mao S, Xiong X. The Interrelation between Reactive Oxygen Species and Autophagy in Neurological Disorders. Oxidative Medicine and Cellular Longevity. 2017;2017:1-16.
